# Hydrostatic pressure improves in-vitro maturation of oocytes derived from vitrified-warmed mouse ovaries

**Published:** 2012-05

**Authors:** Zahra Rashidi, Mehri Azadbakht, Mozafar Khazaei

**Affiliations:** 1 *Fertility and Infertility Research Center, Kermanshah University of Medical Sciences, Kermanshah, Iran.*; 2 *Department of Biology, Faculty of Sciences, Razi University, Kermanshah, Iran.*

**Keywords:** *Vitrification*, *In-vitro maturation*, *Hydrostatic pressure*, *Cell death*, *Mouse*

## Abstract

**Background:** Cryopreservation has limited successes and in-vitro maturation is used to improve its results. Hydrostatic pressure (HP) plays an important role in follicular development.

**Objective:** This study was designed to examine the effects of HP on in-vitro maturation of oocytes and cell death in cumulus cells derived from vitrified-warmed mouse ovaries.

**Materials and Methods:** Preovulatory follicles were harvested from non-vitrified and vitrified-warmed 6-8 week-old female NMRI mouse ovaries and randomly assigned to following groups: non-vitrified (control), non-vitrified with HP exposure (treatment I), vitrified-warmed (treatment II) and vitrified-warmed with HP exposure (treatment III). The follicles of treatments I and III were subjected to HP (20 mmHg) for 30 min and after that all groups were cultured for 24h and assessed for in-vitro maturation of oocytes. The viability and apoptosis of cumulus cells and oocytes were assessed using supravital nuclear staining and TUNEL assay, respectively.

**Results:** Oocytes harvested follicles in both control and treatment II had a significantly lower percentage of metaphase II oocytes (MII) than the treatment I and III (23.5±3.1, 15.03±4.6 and 32.7±3.2, 25.5±4.6; respectively) (p<0.05). Viability of the cumulus cells reduced in treatment I, II and III (83.4, 83.3 and 77.7%) compared to control (86.9%), (p<0.05). The apoptotic index in cumulus and oocyte complexes in treatments I and III (10.7±0.8 and 15.3±0.8) was higher than in control and treatment II (6.7±0.5 and 9.7±0.5) (p<0.05).

**Conclusion:** These results demonstrate that HP had a mild effect on cell death incidence in cumulus cells without any effect on oocyte. However, it can be used as a mechanical force to improve in-vitro maturation of oocytes derived from vitrified-warmed mouse ovaries.

## Introduction

In-vitro maturation (IVM), in-vitro fertilization (IVF) and finally normal offspring have been reported in many studies after cryopreserving of ovarian tissue (1,2). Previous experience found limited success in oocyte cryopreservation. It means few available options for infertility treatment (3). IVM has a long way to success; there are only a few reports of limited success using in-vitro culture of large preantral follicles that have progressed further development (4, 5). 

The cryopreserved follicles have the potential to develop in-vitro; however, the developmental rate is lower than fresh ovarian tissue (6). Researchers have used different cryoprotectants (7, 8), and various techniques to improve the cryopreservation of ovaries (9, 10). Different strategies have been proposed to increase the post-thawing quality of vitrified-warmed ovarian tissue such as inclusion of vitamin A (11), and improvement of environmental and physical condition of follicles (12, 13). These strategies are the most important factors that affect the success of IVM. 

Oocyte maturation is defined as the transition from germinal vesicle (GV) stage, diploten of prophase of the first meiotic division, via germinal vesicle break down (GVBD) to metaphase II (MII) when oocyte can be fertilized with spermatozoid. In the physiological condition, this maturation process depends on several abiotical and biotical parameters and many experiments are being done for optimization of in-vitro conditions, which should be an imitation of in-vivo conditions. In this sense, abiotic parameters are temperature (14), pH value (15), osmotic and hydrostatic pressures (12, 16) and inorganic substances in medium (17), while the most important biotic parameters are organic substances in medium (18), and activators and inhibitors of IVM (19).

Ovarian physical conditions affect follicle rupture and ovulatory process by increasing intrafollicular pressure due to the increase of HP in ovarian vascular system (20). HP is a crucial component of the cellular milieu (21). A decrease of tensile strength of a follicle wall, an increase of inside pressure of follicle, and a combination of these two events lead to the successful rupture of selected follicles. Previous studies have shown a relatively constant intrafollicular pressure, between 15-20 mm Hg, during the entire ovulatory process (20, 22). 

HP, unlike all other parameters, acts immediately and uniformly at each point of in-vitro production (IVP) and it can be applied with the highest precision, consistency, and reliability to mimic in-vivo condition. It has been reported that a well-defined sub lethal high hydrostatic pressure (HHP) treatment offers a solution to improve the overall quality of gametes and embryos, fertilizing ability, and developmental competence (13). In this regard, Du *et al* (12) showed that pre-treatment HHP could considerably improve the IVP of porcine vitrified oocytes. HP has been demonstrated to induce cell death (21) and apoptosis plays a pivotal role during follicular development. 

The aim of present study was to determine the effects of HP on apoptosis in cumulus and oocyte complexes (COCs) and in-vitro maturation of mouse oocyte derived from preovulatory follicles of vitrified-warmed ovarian tissues.

## Materials and methods

**Animals and ovarian tissue**

The present study was reviewed and approved by the Laboratory Animal Care Committee of Kermanshah University of Medical Sciences, Kermanshah, Iran. The 6-8 week female NMRI mice (n=75) were kept at the temperature of 22-24^o^C and 50% humidity in a light-controlled condition (12-h light/12-h dark) and provided with food and water ad libitum. Animals were sacrificed by cervical dislocation, and their ovaries were dissected and allocated randomly categorized into two non-vitrified and vitrified-warmed groups. 

**Experimental design**

To investigate whether HP has effect on the IVM of oocytes, follicles were allocated and cultured in completely randomized design with 4 experimental groups: (i) control: the non-vitrified follicles received no exposure to HP, (ii) treatment I: the non-vitrified follicles were exposed to HP, (iii) treatment II: the vitrified-warmed follicles were not exposed to HP, and (iv) treatment III: the vitrified-warmed follicles were exposed to HP. Consequently, the four groups were assessed for IVM of mouse oocytes, viability of COCs and detection of apoptosis in COCs. Maturation group was repeated 7 times and the other groups were repeated 5 times. 

**Vitrification and warming**

All chemicals were purchased from Sigma-Aldrich (Hamburg, Germany), unless otherwise stated. The vitrification procedure was based on the method reported previously (23) with some modification. Briefly, ovaries were cut into half with a surgical blade and were transferred into the equilibration solution consisting of 7.5% dimethylsulfuxide (DMSO) and 7.5% ethylene glycol (EG) in alpha-Minimal Essential Medium (α-MEM; Gibco), supplemented with 10%. Fetal bovine serum (FBS; Gibco) at room temperature for 15 minutes, and then were transferred into the vitrification solution consisting of 15% DMSO, 15% EG and 0.5 M sucrose dissolved in α- MEM and 20% FBS at 4^o^C for 30 minutes. Then ovaries were placed in the 0.5 ml plastic straw (I.V.M. L’Aigle, France) with a minimum volume of vitrification medium under nitrogen vapor for 30 seconds, and then plunged into liquid nitrogen for 1 week. Then, the straws were taken out of the liquid nitrogen. 

The cryoprotectants were removed by warming the ovaries and diluting them using a four-step dilution with 500 μl of each dilution solution. In brief, ovaries were submerged into 1 ml of descending concentrations of sucrose (1, 0.5, 0.25 and 0.125 M) at room temperature for 5 minutes. The recovered ovaries were transferred to α-MEM supplemented with 20% FBS in 37^o^C for 30 minutes and then the preovulatory follicles were isolated using a 27-G needle under stereomicroscope (Motic; SMZ-143, Spain). 

**In-vitro maturation of oocytes**

The IVM of oocytes was performed according to the method described previously (24) with some modifications. Preovulatory follicles from all groups were transferred to 20 μl microdrops of maturation medium containing α-MEM supplemented with 10% FBS, 10 ng/ml EGF, 100 mIU/ml rFSH (Sereno) and 7.5 IU/ml HCG (Sereno), under detoxified mineral oil (Sigma), in culture plate 60 mm (Falcon) at 37^o^C, under an atmosphere containing 5% CO_2_ in air for 24 hours. Oocytes were denuded and scored as GV, GVBD, metaphase II (MII) and degenerated (DEG) oocytes. GV oocytes have nucleus and are clear. In GVBD oocytes, nucleus is not visible and disappears; in MII oocytes the first polar body is observed. The shrunk, brown or black and fragmented eggs were shown as destroyed oocytes. 

**Hydrostatic pressure exposure**

In treatment I and III, follicles were transferred to pressure chamber for which an established model has been introduced previously (21), and were subjected to 20 mmHg HP for 30 minutes. While, follicles in control and treatment II groups were transferred to similar pressure chamber for 30 minutes without HP exposure. After depressurization, the culture plates were removed from the pressure chamber and cultured for 24 hours. 

**Supravital nuclear staining of cumulus and oocyte complexes (COCs)**

Hoechst PI nuclear staining has been used routinely for quantitative analysis of cell death. Supravital nuclear staining of COCs was performed according to the method described previously with slight modifications (25). Briefly, the COCs were incubated with cell-permeate dye Hoechst 33258 (10 µg/ml in α-MEM) for 15 min at 37^o^C. Then, they were washed and immediately transferred into cell-dye PI (50 µg/ml in α-MEM) just before microscopy. COCs were visualized using a fluorescent microscope (Olympus IX71; Japan) with excitation filters (460 nm for blue fluorescence, and 560 nm for red fluorescence).

**TUNEL staining**

The TUNEL procedure was used to detect DNA fragmentation in combination with PI counterstaining in order to assess nuclear morphology. Nuclear DNA fragmentation in COCs was detected by the TUNEL method using an in situ cell death detection Kit (Roche Diagnostics Corporation Mannheim, Germany (26). Briefly, COCs were fixed in 4% PBS-buffered paraformaldehyde for 60 min at room temperature and then washed three times in PBS and permeabilized with 0.1% Triton X-100 in sodium citrate for 15 minutes on ice. Before labeling, COCs were washed three times in PBS. 

COCs were placed in 30 µl drops of TUNEL reagent and incubated in the dark for 1h at 37^o^C in a humidified chamber. Again COCs were washed three times in PBS and total cell nuclei were labeled with 20 µg/ml PI for 5 min in the dark chamber. After washing twice in PBS, COCs were fixed, stained and subsequently mounted in glycerol. The stained COCs were observed under a fluorescence microscope (Olympus, Japan). The apoptotic index of the COCs was calculated as the percentage of the apoptotic cells relative to the total number of the cells.

**Statistical analysis**

Comparisons between group means were made using one-way analysis of variance (ANOVA). When an effect was statistically significant (p<0.05), mean comparisons were done by post hoc comparisons with a Tukey HSD multiple comparison test. Results are expressed as mean±SEM. The analysis was carried out using SPSS version 16 (Chicago, IL, USA), and the probability of 0.05 or below was considered to be statistically significant.

## Results

**In-vitro maturation of oocytes**

After 24h culture of follicles in maturation medium, Percentage of GV oocytes was higher in control group (30.8±3.01) compared to treatment I (16.1±2.04), treatment II (22.3±2.6) and treatment III (17.8±1.9) (p<0.05) (Table I). Percentage of MII oocytes was higher in treatment I (32.7±3.2) compared to control (23.5±3.1), treatment II (15.03±4.6) and treatment III (25.5±4.6) (p<0.05) (Table I). 

**Supravital nuclear staining of cumulus and oocyte complexes (COCs)**

**Figure 1:**
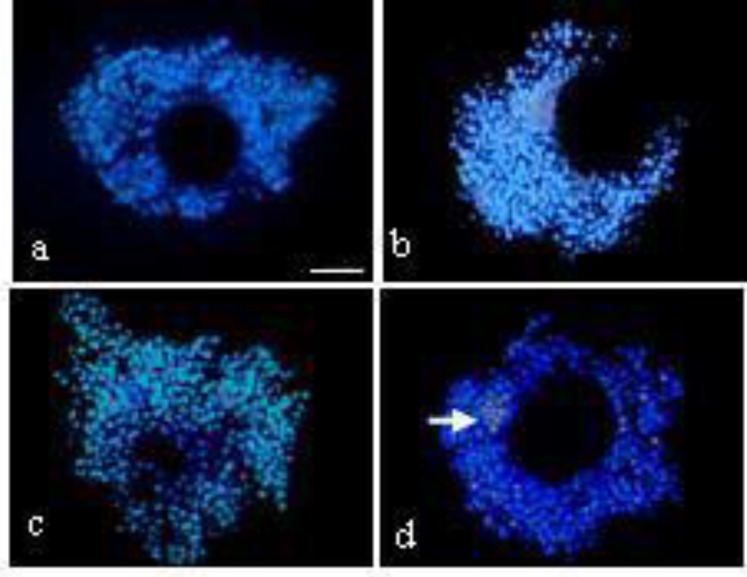
Viability of cumulus and oocytes complex derived from non-vitrified and vitrified-warmed ovaries.

Percentage of viability of cumulus cells after exposure to HP was higher in control (94.8±0.2) compared to treatment I (88.5±0.5), treatment II (85.5±0.7) and treatment III (81.4±0.7) (p<0.05; Table II). After 24h, viability of cumulus cells was higher in control (86.9±0.9) compared to treatment I (83.4±1.0), treatment II (83.3±0.9) and treatment III (77.7±0.5) (p<0.05). There was no significant difference in viability of oocytes among control, treatment I, treatment II and treatment III in 0 and 24 h (Table II) (Figure 1). 

**TUNEL staining**

Cell death was seen in all groups. After pressure exposure, the apoptotic index in cumulus cells was lower in control (2.6±0.3) and treatment II (3.7±0.3) compared to treatment I (5.4±0.4) and treatment III (5.7±0.5) (p<0.05). After 24 h, the apoptotic index in cumulus cells was lower in control (6.7±0.5) compared to treatment I (10.7±0.8), treatment II (9.7±0.5) and treatment III (15.3±0.8) (p<0.05) (Table III) (Figure II).

**Table I:** In-vitro maturation of oocytes derived from non-vitrified and vitrified-warmed ovaries

**Group**	**N**	**GV**	**GVBD**	**MII**	**DEG**
Control	55	30.8 ± 3.01^a^	27.5 ± 5.0 a	23.5 ± 3.1 a	18.1 ± 2.2 a
Treatment I	56	16.1 ± 2.04 b	33.7 ± 2.5 a	32.7 ± 3.2 b	17.5 ± 1.8 a
Treatment II	51	22.3 ± 2.6 a	38.7 ± 6.0 a	15.03 ± 4.6 a	23.8 ± 2.8 a
Treatment III	48	17.8 ± 1.9 b	33.9 ± 3.4 a	25.5 ± 4.6 b	25.1 ± 2.2 a

Control: Non-vitrified, without pressure exposure; Treatment I: Non-vitrified, with pressure exposure; Treatment II: Vitrified-warmed, without pressure exposure; Treatment III: Vitrified-warmed, with pressure exposure; GV: Germinal vesicle; GVBD: Germinal vesicle break down; MII: Metaphase II; DEG: Degenerate oocytes.

a/b Values within columns with different superscripts are significantly different (ANOVA, p < 0.05).

**Table II:** Viability of cumulus and oocytes complex derived from non-vitrified and vitrified-warmed ovaries during in-vitro maturation.

		**Cumulus cells**		**Oocyte**	
				**I (GV)**	**II (MII)**
		**0 h**	**24 h**	**0 h**	**24 h**
**Group**	**N**	**Viable (%)**	**Viable (%)**	**Viable (%)**	**Viable (%)**
Control	25	94.8 ± 0.2 a	86.9 ± 0.9 aa	89.1 ± 0.6 a	85.3 ± 2.6 a
Treatment I	25	88.5 ± 0.5 b	83.4 ± 1.0 ab	88.8 ± 0.5 a	84.8 ± 2.5 a
Treatment II	25	85.5 ± 0.7 c	83.3 ± 0.9 ab	86.0 ± 1.0 a	82.7 ± 0.5 a
Treatment III	25	81.4 ± 0.7 d	77.7 ± 0.5 ac	83.5 ± 1.5 a	80.3 ± 1.2 a

Control: Non-vitrified, without pressure exposure; Treatment I: Non-vitrified, with pressure exposure; Treatment II: Vitrified-warmed, without pressure exposure; Treatment III: Vitrified-warmed, with pressure exposure.

a/b/c/d Values within columns with different superscripts are significantly different (ANOVA, p < 0.05).

**Table III:** Cell death in COCs derived from non-vitrified and vitrified-warmed ovaries

**Group**	**N**	**0h**	**24h**
Control	25	2.6 ± 0.3 a	6.7 ± 0.5 a
Treatment I	25	5.4 ± 0.4 b	10.7 ± 0.8 b
Treatment II	25	3.7 ± 0.3 a	9.7 ± 0.5 c
Treatment III	25	5.7 ± 0.5 b	15.3 ± 0.8^d^

Control: Non-vitrified, without pressure exposure; Treatment I: Non-vitrified, with pressure exposure; Treatment II: Vitrified-warmed, without pressure exposure; Treatment III: Vitrified-warmed, with pressure exposure.

Apoptotic index is the number of cells displaying both TUNEL and condensation of the nuclei as a proportion of the total number of cells in a COCs. Data are means±S.E.M.

a/b/c/d Values within columns with different superscripts are significantly different (ANOVA, p < 0.05).

a: Non-vitrified, without pressure exposure ;

b: Non-vitrified, with pressure exposure;

c: Vitrified-warmed, without pressure exposure;

d: Vitrified-warmed, with pressure exposure. Scale bar: 50µm.

**Figure 2:**
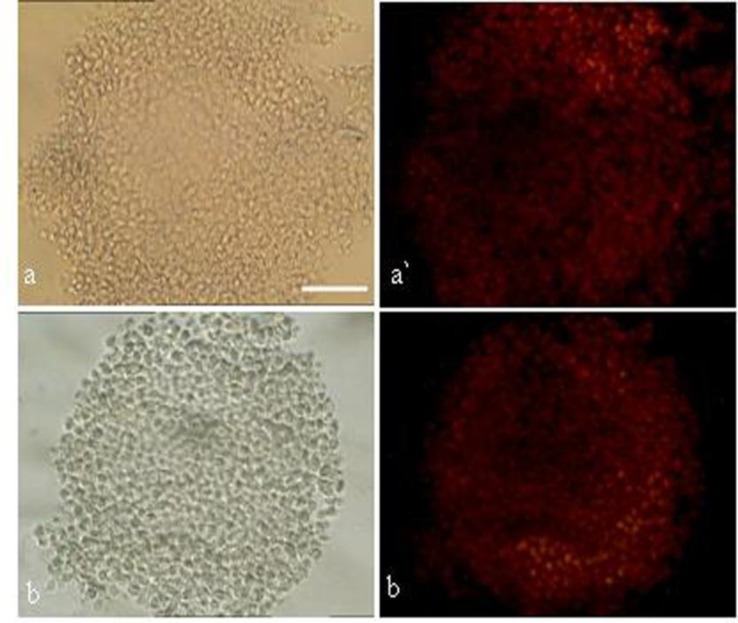
epresentative image of cumulus and oocyte complexes after in-vitro maturation subjected to TUNEL analysis to determine apoptosis.

a, a`: without pressure exposure;

b, b`: with pressure exposure. Scale bar: 50µm.

## Discussion

The current study indicated that the IVM rate in oocytes derived from preovulatory follicles of vitrified-warmed mouse ovarian tissue increased following exposure to HP. This improvement occurred by increasing in COCs apoptosis. However, the viability of oocytes derived from both vitrified-warmed and non-vitrified samples was similar and independent of exposure to HP. HP is a crucial component of the cells environments (21) and is effective in reproductive systems. Intraluminal pressure change has been measured in the oviduct, uterus and cervix of the mated rabbits (27). The effect of HP has been studied on some cells. 

Du *et al* reported improvement of in-vitro developmental competence of porcine mature vitrified oocyte after exposure to 20-40 MPa HP (12), also Pribenszky *et al* introduced the effect of High HP treatment prior to vitrification on the survival of expanded mouse blastocysts and demonstrated that a preceding pressure treatment strikingly increases the survival of the frozen blastocysts as well as the speed of resumption of the development and hatching rate. 

High HP induced stress tolerance in porcine spermatozoa (28, 29), but here we showed the beneficial effect of lower HP on follicular development, also Porcine oocytes were found relatively sensitive to HHP, accordingly a 20 MPa pressure for 60 min proved to be the optimal treatment to increase stress tolerance (30). Cryopreservation by vitrification has been successfully applied to ovarian tissue of mice (8). 

A pervious report showed that only primordial and primary follicles were survived from surgically implanted cryopreserved ovaries (31) and after ovarian transfer, antral follicle didn't persist, because of impaired angiogenesis. Moreover, because of the size and cellular complexity of antral follicles, it is also possible that follicular somatic cells or oocytes become damaged by incomplete permeation of cryoprotectants (32). 

However, oocytes could prepare, matured and fertilized from antral follicles of cryopreserved ovaries (1). There is a relatively constant intrafollicular pressure ranged 15-20 mm Hg during the entire ovulatory process that increases in ovulating process. Since COCs in preovulatory follicles were exposed to intrafollicular pressure during ovulatory process, in line with those observations, 20 mmHg pressures were used (20). 

We isolated preovulatory follicles and exposed them to HP. The percentage of MII oocytes considered as oocyte maturation significantly increased in follicles exposed to HP in comparison to follicles not exposed to HP. These results indicated that HP improved oocytes maturation. In addition to improvement in oocyte maturation, HP increased the cell death in COCs derived from non-vitrified and vitrified-warmed follicles. Cumulus cells play a critical role in oocyte maturation and fertilization. Cumulus cells dissociate during the ovulatory process and ovum is released into the follicular fluid antrum (33). Also, it is suggested that moderate apoptotic changes in follicles might even support or induce prematuration-like changes of oocytes which are typically necessary for their preovulatory development (34). In another study, Ikeda *et al* demonstrated that cumulus cells in bovine cumulus-enclosed oocytes spontaneously undergo apoptosis during IVM (35). 

There are several reports about the effects of cryopreservation on the incidence of apoptosis in ovarian tissue after thawing (36-38). Investigation of apoptotic cell death with TUNEL staining has been shown in bovine COCs (24, 39). In this study, the percentage of apoptotic cells significantly increased in follicles after exposure to HP without exerting any effects on viability of oocytes. Therefore, HP as a cell death inducer (21) increased the incidence of apoptotic cells in COCs derived from vitrified-warmed and non-vitrified ovaries and the rate of oocyte maturation increased without any changes in oocytes viability.

## Conclusion

In conclusion, the present study indicated that HP enhances the IVM of the oocytes from non-vitrified and vitrified-warmed ovaries. Improving maturation by hydrostatic pressure in the present experiment resulted in an increase in cell death incidence in cumulus cells with no signs of cell death in mouse oocyte. Additional studies are required to find the mechanism that may lie beyond this observation.

## References

[1] Sztein JM, O'Brien MJ, Farley JS, Mobraaten LE, Eppig JJ (2000). Rescus of oocytes from antral follicles of cryopreservation mouse ovaries: competence to undergo maturation, embryology and development to term. Hum Reprod.

[2] Newton H, Illingworth P (2001). In-vitro growth of murine pre-antral follicles after isolation from cryopreserved ovarian tissue. Hum Reprod.

[3] Fabbri R, Porcu E, Marsella T, Rocchetta G, Venturoli S, Flamigni C (2001). Human oocyte cryopreservation: new perspectives regarding oocyte survival. Hum Reprod.

[4] Abir R, Roizman P, Fisch B, Nitke S, Okon E, Orvieto R (1999). Pilot study of isolated early human follicles cultured in collagen gels for 24 hours. Hum Reprod.

[5] Wu J, Emery BR, Carrell DT (2001). In-vitro growth, maturation, fertilization, and embryonic development of oocytes from porcine preantral follicles. Biol Reprod.

[6] Segino M, Ikeda M, Hirahara F, Sato K (2005). In-vitro follicular development of cryopreserved mouse ovarian tissue. Reproduction.

[7] Rodrigues AP, Amorim CA, Costa SH, Matos MH, Santos RR, Lucci CM (2004). Cryopreservation of caprine ovarian tissue using glycerol and ethylene glycol. Theriogenology.

[8] Rodrigues AP, Amorim CA, Costa SH, Matos MH, Santos RR, Lucci CM (2004). Cryopreservation of caprine ovarian tissue using dimethylsulphoxide and propanediol. Anim Reprod Sci.

[9] Hasegawa A, Mochida N, Ogasawara T, Koyama K (2006). Pup birth from mouse oocytes in preantral follicles derived from vitrified and warmed ovaries followed by in-vitro growth, in-vitro maturation, and in vitro fertilization. Fertil Steril.

[10] Kagawa N, Kuwayama M, Nakata K, Vajta G, Silber S, Manabe N (2007). Production of the first offspring from oocytes derived from fresh and cryopreserved pre- antral follicles of adult mice. Reprod Biomed Online.

[11] Babaei H, Nematallahi-Mahani SN, Kheradmand A (2006). The effects of Vitamin A administration on the development of vitrified-warmed mouse blastocyst. Anim reprod.

[12] Du Y, Pribenszky CS, Molnar M, Zhang X, Yang H, Kuwayama M (2008). High hydrostatic pressure: a new way to improve in vitro developmental competence of porcine matured oocyte after vitrification. Reproduction.

[13] Pribenszky C, Vajta G (2011). Sublethal hydrostatic pressure treatment of gametes and embryos: a fundamentally new approach for improving assisted reproductive techniques. Reprod Fertil Dev.

[14] Sugiyama S, Mcgowan M, Phillips N, Kafi M, Young M (2007). Effects of increased ambient temperature during IVM and/or IVF on the in vitro development of bovine zygotes. Reprod Domes Anim.

[15] Smiljakovic T, Josipovic S, Kosovac O, Delic N, Aleksic S, Petrovic MM (2008). The role of pH values in porcine reproductive tracts of male and female individuals. Biotech Anim Husband.

[16] Van Den Abbeel E, Schneider U, Liu J, Agca Y, Critser JK, Van Steirteghem A (2007). Osmotic responses and tolerance limits to changes in external osmolalities, and oolemma permeability characteristics of human in vitro matured MII oocytes. Hum Reprod.

[17] Yamauchi N, Sasada H, Sugawara S (1995). In-vitro maturation of porcine follicular oocyte using medium-II. Tohoku Agric Res.

[18] Smiljaković T, Sretenović Lj, Aleksić S (2009). Influence of abiotic and biotic factors on maturation of oocytes (mammalian eggs) in-vitro conditions. Biotech Anim Husband.

[19] Smiljaković T, Tomek W (2006). Meiotic maturation and in vitro maturation of bovin oocytes. Biotech in Anim Husb.

[20] Matousek M, Carati C, Gannon B, Brännström M (2001). Novel method for intrafollicular pressure measurements in the rat ovary: increased intrafollicular pressure after hCG stimulation. Reproduction.

[21] Agar A, Li Sh, Agarwal N, Coroneo MT, Hill MA (2006). Retinal ganglion cell line apoptosis induced by hydrostatic pressure. Brain Res.

[22] Talbot P (1983). Intrafollicular pressure promotes partial evacuation of the antrum during hamster ovulation in-vitro. J Exp Zool.

[23] Eimani H, Safari Mamzoji S, Soleimani Mehranjani M, Abnosic MH, Rezazadeh Valojerdi M, Eftekhari Yazdi P (2007). Survival rate of preantral follicles derived from vitrified neonate mouse ovarian tissue by cryotop and conventional methods. Bio Factors.

[24] Pesty A, Miyara F, Debey P, Lefevre B, Poirot C (2007). Multiparameter assessment of mouse oogenesis during follicular growth in-vitro. Mol Hum Reprod.

[25] Lu D, Bai XC, Gui L, Su YC, Deng F, Liu B (2004). Hydrogen peroxide in the Burkitt's lymphoma cell line Raji provides protection against arsenic trioxide-induced apoptosis via the phosphoinositide-3 kinase signalling pathway. Br J of Haematol.

[26] Pocar P, Nestler D, Risch M, Fischer B (2005). Apoptosis in bovine cumulus-oocyte complexes after exposure to polychlorinated biphenyl mixtures during in-vitro maturation. Reproduction.

[27] Suzuki H, Tsutsumi Y (1981). Intraluminal pressure changes in the oviduct, uterus, and cervix of the mated rabbit. Biol Reprod.

[28] Pribenszky C, Molnar M, Cseh S, Solti L (2005). Improving post-thaw survival of cryopreserved mouse blastocysts by hydrostatic pressure challenge. Anim Reprod Sci.

[29] Pribenszky C, Molnar M, Horvath A, Harnos A, Szenci O (2005). Hydrostatic pressure induced increase in post-thaw motility of frozen boar spermatozoa. Reprod Fertil Dev.

[30] Pribenszky C, Du Y, Moln´ar M, Harnos A, Vajta G (2008). Increased stress tolerance of matured pig oocyte by high hydrostatic pressure impulse. Anim Reprod Sci.

[31] Sztein J, Sweet H, Forley J, Mobrraten L (1998). Cryopreservation and orthotopic transplantation of mouse ovaries: new approach in gamete banking. Biol Reprod.

[32] Harp R, Leibach J, Black J (1994). Keldahl Karow A. Cryopreservation of murine ovarian tissue. Cryobiology.

[33] Murdoch WJ, Gottsch ML (2003). Proteolytic mechanisms in the ovulatory folliculo-luteal transformation. Connect Tissue Res.

[34] Hendriksen PJM, Vos PLAM, Steenweg WNM, Bevers MM, Dieleman SJ (2000). Bovine follicular development and its effect on the in-vitro competence of oocyte. Theriogenology.

[35] Ikeda S, Imai H, Yamada M (2003). Apoptosis in cumulus cells during in vitro maturation of bovine cumulus-enclosed oocytes. Reproduction.

[36] Demirci B, Salle B, Frappart L, Frank M, Guerin JF, Lornage J (2002). Morphological alterations and DNA fragmentation in oocytes from primordial and primary follicles after freezing-thawing of ovarian cortex in sheep. Fertil Steril.

[37] Bedaiwy MR, Hossein MR (2004). Histological evaluation and in situ localization of apoptosis in fresh and cryopreserved ovarian tissue. Middle East Fertil Soc.

[38] Rimon E, Cohen T, Dantes A, Hirsh L, Amit A, Lessing JB (2005). Apoptosis in cryopreserved human ovarian tissue obtained from cancer patients: a tool for evaluating cryopreservation utility. Int J Oncol.

[39] Yuan YQ, Van Soom A, Leroy JLMR, Dewulf J, Van Zeveren A, Kruif AD (2005). Apoptosis in cumulus cells, but not in oocytes, may influence bovine embryonic developmental competence. Theriogenology.

